# Granuloma debridement and the use of an injectable calcium phosphate bone cement in the treatment of osteolysis in an uncemented total knee replacement

**DOI:** 10.1186/1749-799X-5-29

**Published:** 2010-04-27

**Authors:** Henry D Atkinson, Vijai S Ranawat, Roger D Oakeshott

**Affiliations:** 1 Department of Trauma and Orthopaedics and North London Sports Orthopaedics, North Middlesex University Hospital, Sterling Way, London N18 1QX, UK; 2 Sportsmed SA, 32 Payneham Road, Stepney, Adelaide 5069, Australia

## Abstract

Polyethylene particulate debris-induced periprosthetic osteolysis is a known complication of knee arthroplasty surgery, and may result in the need for revision surgery. The management of these bony defects can be surgically challenging, and full revisions of well-fixed total knee components can lead to substantial bone loss. We present the case of a 71 year old man who developed knee pain and osteolysis around an uncemented total knee replacement. Due to significant medical comorbidies he was treated by percutaneous cyst granuloma debridement and grafting using an injectable calcium phosphate bone substitute. There were no wound complications, and the patient was allowed to fully weight-bear post-operatively. Histopathology and microbiology of the cyst material confirmed polyethylene granulomata without any evidence of infection. At 6 weeks post-operatively the patient's previous knee pain had resolved, he was able to comfortably fully weight-bear. Preoperative scores (Knee Society Score (KSS) 41, WOMAC score 46.2, and Oxford Knee Score 39) had all improved at the 12-month post-operative review KSS 76, WOMAC 81.7 and Oxford Knee score 21). This is a safe and effective technique with minimal morbidity and may be an appropriate treatment modality when more extensive revision surgery is not possible. The case is discussed with reference to the literature.

## Background

Polyethylene particulate debris-induced periprosthetic osteolysis is a known complication of knee arthroplasty surgery, and may result in the need for revision surgery. The management of these bony defects can be surgically challenging, and full revisions of well-fixed total knee components can lead to substantial bone loss.

Granuloma debridement and grafting of osteolytic defects around well-fixed cementless implants has been successfully performed in total hip arthroplasty [1-7]; and recovery can be relatively quick as these osseointegrated implants do not require postoperative activity restriction [8]. Allograft bone chips are typically used, and though a variety of bone substitutes have also been utilised, their efficacy has not been widely documented.

We present the case of a patient with osteolysis occurring around an uncemented total knee replacement, treated by cyst granuloma debridement and grafting using an injectable calcium phosphate bone substitute.

## Case Report

In May 1996, a 59 year-old moderately obese farmer had 1 week-staged uncemented bilateral total knee replacements without patella resurfacing. He had severe medial and lateral compartment osteoarthritis with minimal osteophytes on the patella, and had previously undergone arthroscopic debridement procedures in both knees 2 years earlier. He made a good post-operative recovery, with 0-110 degree active flexion by 6 weeks, normal lower limb alignment and a resolution of knee pain symptoms, returning to work at 3 months.

In May 1997 he began to develop increasing bilateral anterior knee pain especially while walking on stairs and inclines. Radiographs showed progression in the patellar osteoarthritis and a bone scan revealed increased uptake in both patellae. He underwent bilateral synchronous patellar resurfacings with cemented components in July 1997. There was no significant wear seen on either tibial polyethylene spacer, however as there was florid synovitis in both knees, the tibial spacers were exchanged and the patient also had total synovectomies. Post-operatively he recovered well with 0-110 knee flexion having been re-gained by 3 months post-revision surgery.

However, in August 1998 he began developing some medial tibial pain and mild swelling in the right knee, which continued and by August 2000 had developed large granulomatous cysts around the stem of the tibial component. He underwent revision surgery on the right knee in October 2000. Intraoperatively, he was once again found to have massive synovitis and so underwent a total synovectomy. There was a 3 × 3 cm uncontained bony perforation medial to the tibial tubercle, and the tibial polyethylene had badly delaminated and was excessively worn. The tibial component was removed and the granulomatous material removed from the proximal tibia. The femoral component, which was poorly ingrown, was also removed leaving an anterior cortical defect. The femoral and tibial bony cortical defects were reconstructed with anatomic specific allograft and the cavities impaction grafted with morcelised bone. The knee was then revised using stemmed cemented implants. Histolopathology and microbiological analyses confirmed polyethylene granulomata, polyethylene particulate debris and synovitis, with no evidence of infection. The bone graft incorporated fully by 9 months and the right knee remains pain-free with good function and a 0-125 degree range of flexion 8 years later.

In January 2005 the patient began to develop similar medial pain and swelling in the left knee. A bone scan showed increased activity in the medial femoral condyle indicating possible localised osteolysis, and there was some low-grade uptake in the proximal tibia and suprapatellar pouch, though no abnormalities were seen on CT. The patient underwent a left knee arthroscopy in May 2005, where he was once again found to have florid synovitis though the patellar button and tibial spacer appeared to be in a good condition. He underwent a full synovectomy and had good post-operative clinical improvement with a resolution of the knee swelling and pain by 3 months.

**Figure 1:**
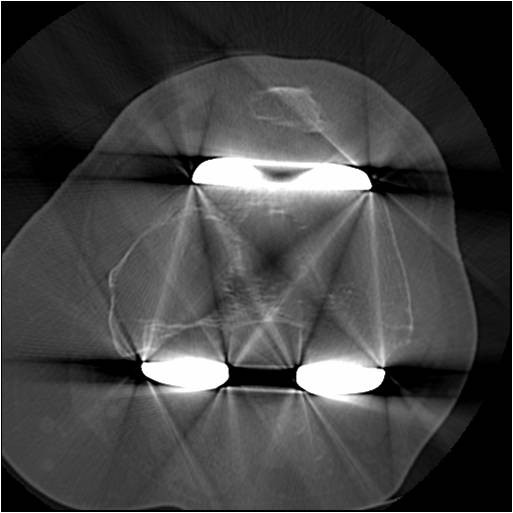
**Axial CT of Femur demonstrating large lytic area in the medial femoral condyle**.

**Figure 2:**
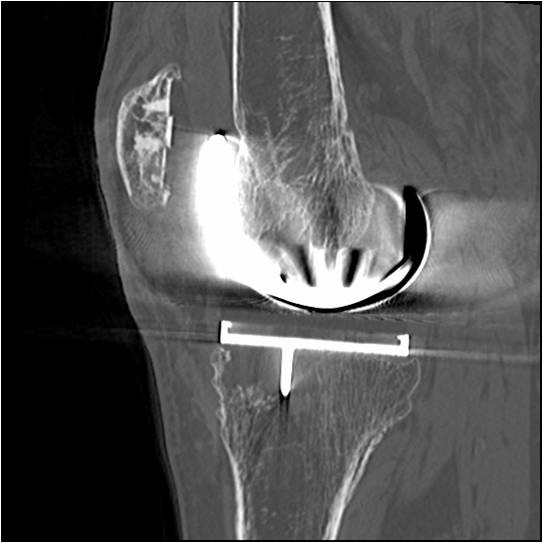
**Sagittal CT demonstrating the lytic area in the antero-medial aspect of the tibia**.

He remained symptom-free until 2008, when he began developing medial pain in the left knee and difficulty weight-bearing. His Knee Society Score (KSS) was 41, WOMAC score was 46.2, and Oxford Knee Score 39. Clinically there was no knee swelling or effusion, and he did not appear to have active synovitis; the symptoms appeared to be more characteristic of implant loosening. Laboratory screening including erythrocyte sedimentation rate and C-reactive protein was normal. Plain radiographs revealed areas of periprosthetic osteolysis in the femur and the tibia, and a CT scan demonstrated an extensive lytic area measuring 16 cm^3 ^in the medial femoral condyle and a 3.6 cm^3 ^cyst in the medial tibial condyle; both consistent with polyethylene particulate disease (Figures 1 and 2). A further bone scan did not indicate any clear evidence of component loosening, and successive knee radiographs had not shown obvious joint-space narrowing. Nevertheless with this amount of bone loss major revision knee surgery, similar to that performed on the right knee, appeared to now be clinically and radiologically indicated in the left knee. Unfortunately over this period the patient had developed renal failure and had undergone renal transplant surgery in December 2005. He required daily oral steroid and immunosuppressive therapy, and unfortunately still had poor renal function. His renal physicians advised against any further major surgery. It was thus decided to perform a curettage and grafting of these femoral and tibial bony defects alone, to improve the patient's pain symptoms and to halt any cyst progression, and subsequent fracture or catastrophic implant failure.

**Figure 3:**
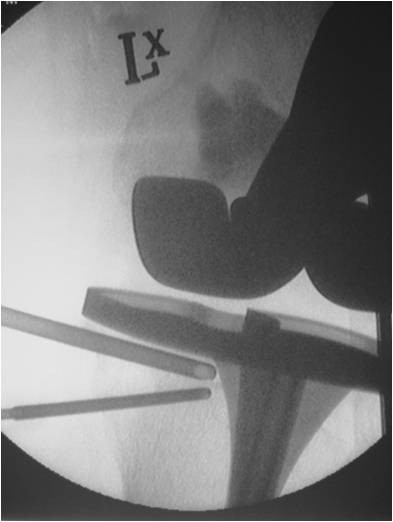
**Fluoroscopic image of the left knee showing the introducing cannula and simultaneous venting of the medial tibial condylar cyst**.

**Figure 4:**
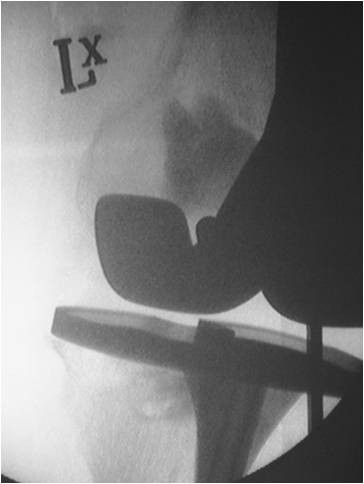
**Fluoroscopic image of the left knee after cementation of the medial femoral and medial tibial condyles**.

**Figure 5:**
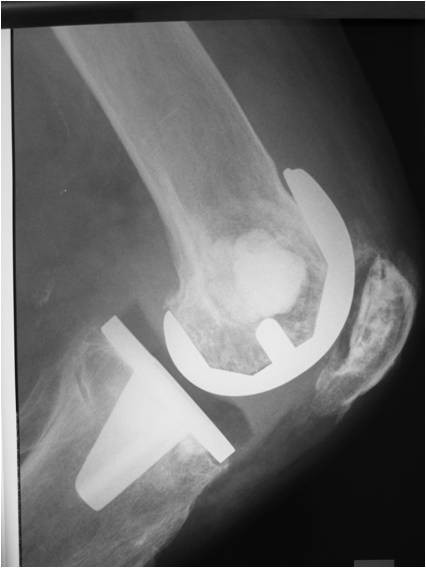
**Lateral radiograph of the knee demonstrating the area of calcium phosphate bone cementage in the medial femoral and medial tibial condyles**.

**Figure 6:**
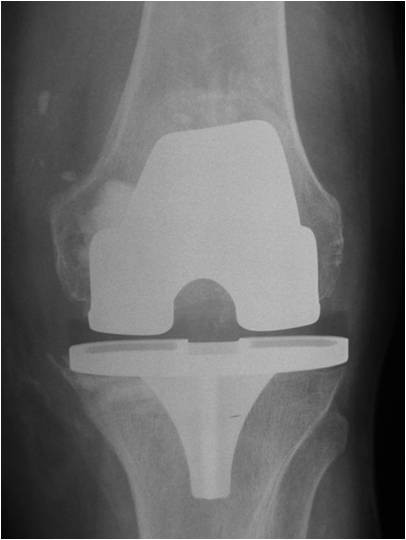
**AP radiograph of the knee demonstrating the area of calcium phosphate bone cementage in the medial femoral and medial tibial condyles**.

In December 2008, under a short general anaesthetic, two 3 cm incisions were made over both the medial femoral and medial tibial condyles. Two 4.5 mm drill holes were made in both defects under image intensification, and an arthroscopic chondrotome was introduced into each defect to clear it of its granulomatous content. Hydroset™ (Stryker Howmedica) calcium phosphate was then inserted under direct vision into both defects, with simultaneous venting to allow for free inflow of the material (Figures 3 and 4). After 8 minutes the calcium phosphate had set, and the wounds were closed routinely without drains. There were no wound complications, or blood chemistry derangements, and the patient was allowed to fully weight-bear post-operatively. Histopathology and microbiology of the cyst material confirmed polyethylene granulomata, without any evidence of infection. At 6 week review the patient's knee pain had resolved, and he was able to comfortably fully weight-bear. The graft material showed some signs of incorporation on x-ray by 4 months (Figures 5 and 6), and the patient remains well at their 12 month review. KSS at most recent view was 76, WOMAC score 81.7 and Oxford Knee score 21. The patient will continue to have regular clinical and radiological implant surveillance.

## Discussion

When dealing with massive periprosthetic osteolysis the aims of surgery are usually to restore bone stock around the arthroplasty, gain stable implant fixation, restore the joint mechanics and reduce the particle debris load [8]. This was successfully accomplished in the patient's contralateral knee which underwent a full revision knee arthroplasty with stemmed implants and extensive bone-grafting in 2000, following severe polyethylene delamination, osteolysis and component loosening. However, treatment decisions are also very much dependent on non-joint patient factors, such as the patient's clinical status and physiological age, comorbidities, and activity levels [8-11], and our patient was deemed no longer medically fit to undergo any kind of extensive or invasive surgery. He thus underwent minimally invasive cyst debridement and grafting alone.

One can argue that the cyst debridements should have been performed in conjunction with a further total synovectomy and tibial polyethylene spacer exchange, in order to remove any residual particulate material and the wear particle generator [8]. Indeed modular polyethylene exchange is a reasonable option when dealing with polyethylene wear and osteolysis in patients with well-fixed knee components [12,13], without evidence of accelerated wear or severe delamination [10], and has similar short-term results and failure rates when compared with patients having full revisions of all the knee components [8]. However, our patient had an arthroscopic synovectomy almost 4 years earlier during which no visible polyethylene delamination or macroscopic wear was seen, and successive knee radiographs had not shown progressive joint space narrowing. Additionally, the patient's presenting symptoms were not consistent with active synovitis, and the authors were keen to avoid the more extensive surgery required for a bearing exchange that may not have been necessary.

Traditionally, bony defects in revision knee arthroplasty have been treated with autologous bone, allograft, polymethylmethacrylate (PMMA) bone cement and implant augments [9-11,14], however not all these options were open to our patient. Given the minimally invasive nature of the intended surgery, we considered using PMMA as a void filler for our patient's osteolytic cysts. Cementation would have had the advantage of providing immediate stability [15,16], though would not have functioned as a biological scaffold, and might have caused thermal necrosis of the surrounding bone possibly leading to further osteolysis [17,18]. Autologous bone grafting was also considered because of its biological advantages, as well as its proven record in dealing with small tibial bony defects [19], and with impaction grafting in knee and hip revision arthroplasty [20].

However we decided instead to use Hydroset™, an injectable osteoconductive calcium phosphate bone cement, for its versatility and simplicity of use [21], and to avoid any additional donor-site morbidity. Hydroset™ is composed of a mixture of tetracalcium phosphate, dicalcium phosphate dihydrate and trisodium citrate, which crystallizes to form hydroxyapatite without an exothermic reaction, reaching 75% compressive strength by 4 hours and full strength at 24 hours [21]. It physically interdigitates with the adjacent bone [21], and though not designed to provide any structural support, forms a structure that is more mechanically stable than either cancellous bone graft or bone substitute blocks or pellets [22-24]. It has improved manual handling and mechanical properties when compared with other calcium phosphate and calcium sulphate cements [21,25,26] and creates a scaffold for osteogenesis which is gradually replaced by creeping substitution [21].

We were unable to find any data in the literature pertaining to the use of bone cements in the context of osteolytic cysts in knee arthroplasty, however these analogues have been successfully used in the treatment of benign bone tumors, and for hardware augmentation in fracture surgery, and have proven biocompatibility, bioactivity and osteoconductivity [26-30] One study of 13 patients with large expansive (mean volume of 38.5 mls) osteolytic benign bone tumors, (similar in size to our patient's cysts) used composite bioceramic osteoconductive grafts, combining porous hydroxyapatite with calcium sulphate, following curettage and phenolisation. 11 of the13 lesions displayed clinical and radiological consolidation at a mean of 4.6 months, and patients had good return to normal function [31]. Another study of 23 patients with bone cysts or benign bone tumors (measuring a mean of 23 mls) treated with calcium-sulphate pellets with or without added demineralised bone matrix, found complete bone regeneration by 6 months in both treatment groups [32]. A multicenter trial of 46 patients with benign bone tumors (ranging in size from 0.15 to 112 mls), found 96% bony ingrowth and 100% graft resorption at 12 months when using the same calcium-sulphate pellets, with no difference between those patients treated with calcium-sulphate alone or in combination osteoinductive agents [33]. Additionally, a recent meta-analysis of 14 randomized controlled trials using calcium phosphate bone cement for augmentation of metaphyseal fracture fixation (in the tibial plateau, femoral neck and calcaneum), found that patients had a lower prevalence of pain at the fracture site and a decrease in the loss of fracture reduction, compared with those patients treated with autogenous bone graft [34].

## Conclusion

Our patient had no intraoperative or postoperative complications. He has had a dramatic improvement in KSS, WOMAC and Oxford Knee scores and remains well and fully ambulatory at 12 months. We believe that injectable osteoconductive calcium phosphate bone cements may be a useful adjunct in treating osteolytic cysts around well-fixed knee replacement components.

## Abbreviations

WOMAC: Western Ontario and McMaster osteoarthritis index; CT: Computed Tomography; KSS: Knee Society Score; PMMA: polymethylmethacrylate.

## Consent

Written informed consent was obtained from the patient for publication of this case report and any accompanying images. A copy of the written consent is available for review by the Editor-in-Chief of this journal

## Competing interests

The authors declare that they have no competing interests.

## Authors' contributions

RO operated the patient and is the senior author. HA managed the patient and wrote the manuscript. VR assisted with the literature review and manuscript preparation.

All authors have read and approved the final manuscript.

## Author information

Henry D Atkinson, MBChB, BSc, FRCS Tr & Orth

Department of Trauma and Orthopaedics and North London Sports Orthopaedics, North Middlesex University Hospital, Sterling Way, London N18 1QX, UK.

and: Sportsmed SA, 32 Payneham Road, Stepney 5069, Adelaide, South Australia.

Vijai S Ranawat, MBBS, FRCS Tr & Orth

Department of Trauma and Orthopaedics, The Whittington Hospital, Highgate Hill, Archway, London N19 5NF, UK.

and: Sportsmed SA, 32 Payneham Road, Stepney 5069, Adelaide, South Australia.

Roger D Oakeshott, MBBS, FAOrthA, FRACS

Sportsmed SA, 32 Payneham Road, Stepney 5069, Adelaide, South Australia.
